# Surrogacy relationships: a critical interpretative review

**DOI:** 10.1080/03009734.2020.1725935

**Published:** 2020-02-19

**Authors:** Jenny Gunnarsson Payne, Elzbieta Korolczuk, Signe Mezinska

**Affiliations:** a Department of Historical and Contemporary Studies, Sodertorns Hogskola, Huddinge, Sweden;; b Faculty of Medicine, University of Latvia, Riga, Latvia

**Keywords:** Assisted reproduction, critical interpretative review, ethnography, qualitative interviews, qualitative methods, relational ethics, reproductive justice, surrogacy, surrogate motherhood

## Abstract

Based on a critical interpretative review of existing qualitative research investigating accounts of ‘lived experience’ of surrogates and intended parents from a relational perspective, this article proposes a typology of surrogacy arrangements. The review is based on the analysis of 39 articles, which belong to a range of different disciplines (mostly sociology, social psychology, anthropology, ethnology, and gender studies). The number of interviews in each study range from as few as seven to over one hundred. Countries covered include Australia, Canada, Greece, India, Iran, Israel, Italy, Mexico, Norway, Russia, Sweden, UK, Ukraine, and the USA. Most studies focus only on surrogacy practices in one country (although often with intended parents from other countries), and some include several countries (e.g. interviewees from several countries or fieldwork in different field-sites). The proposed typology goes beyond the division between altruistic versus commercial, and traditional versus gestational surrogacy, in order to inform further research and to contribute to bioethical and policy debates on surrogacy in a transnational context. Four types of relations are identifiable: open, restricted, structured, and enmeshed. The criteria which influence these relationships are: the frequency and character of contact pre- and post-birth; expectations of both parties; the type of exchange involved in surrogacy arrangements; and cultural, legal, and economic contexts. The theoretical contribution of the article is to further the development of a relational justice approach to surrogacy.

## Introduction and background

Surrogacy, the situation where a person intentionally gets pregnant and carries a child for someone else, began flourishing in the USA in the 1980s, and today it has grown into a global trend. Sometimes arrangements are non-paid (altruistic), but often they are contracted and paid (commercial). In traditional or partial surrogacy, the surrogate’s own eggs are used, hence making the surrogate and the surrogacy-conceived child genetically related. In gestational or full surrogacy, the egg of either the intended mother or an egg-donor is used. Partial surrogacy was more common in the earlier phase of contemporary surrogacy (1), but in contemporary surrogacy practices it is far more common to use the eggs from one of the intended parents or from a donor, often anonymous. Like other forms of collaborative reproduction, surrogacy involves new parties in the process of reproduction and creates new relationships, leading to questions about emotions, rights, and responsibilities of all people involved.

In current debates, there are strong disputes whether surrogacy strengthens reproductive rights (e.g. women’s right to choose, involuntarily childless people’s reproductive rights, LGBTQ rights) or impedes them (e.g. surrogates’ right to abortion or to keep the child). As such, the ethical and political debates on surrogacy are intrinsically intertwined not only with medical and psychological risks, but also with issues concerning autonomy, agency, and justice, as well as risks of coercion and exploitation. Moreover, the laws regulating surrogacy differ widely between countries.

## Research question and aim

Through critical review of existing qualitative research, this article seeks to explore the possibilities of developing a relational justice approach to surrogacy. Specifically, we seek to investigate existing accounts of the ‘lived experience’ of surrogates and intended parents from a relational perspective, focussing in particular on the way surrogates and intended parents narrate the relationship to each other and the child. The goal is to propose a typology of surrogacy arrangements going beyond the division between altruistic versus commercial and traditional/partial versus gestational surrogacy, in order to inform further research and to contribute to bioethical and policy debates on surrogacy in a transnational context.

Thereby, we seek to identify under what circumstances the reproductive parties themselves describe the relationship as satisfactory, fair, and just, and by extension what experts and lawmakers need to take into account when debating not only whether to permit surrogacy or not, but also how a framework of relational justice might be formulated in a way that takes the rights, well-being, and emotions of all reproductive parties equally into account throughout the surrogacy process and after the birth.

## Previous research

While there are no official global statistics, it is well established that surrogacy has recently increased exponentially (2), as has research in this field. Even though there are a few notable exceptions, review articles analysing the effects of surrogacy on the surrogate, intended parents, and children are still remarkably few, and the relational aspect of surrogacy remains surprisingly understudied.

Söderström-Anttila et al. (3) have conducted a systematic review focussing on obstetric, medical, and psychological outcomes for surrogates, surrogacy-conceived children, and their parents. Only 55 out of 1795 studies were deemed of sufficient quality for review. Their review concludes that obstetric, medical, and psychological outcomes in existing studies show no significant difference between surrogacy pregnancies and other pregnancies, but argues that due to lacking numbers and quality of studies results should be interpreted with caution. Van den Akker (4) points out strongly selective samples in existing studies but shows that research indicates that openness and disclosure to surrogacy-conceived children about their conception are common, and that most intended parents intend to keep in touch with surrogates after birth. The study also discusses how in the UK, where much of the research is carried out, it is common for intended parents and surrogates to develop a close relationship during the surrogacy process. Edelman (5) points out the lack of research but concludes that surrogacy generally seems to be a positive experience for everyone involved, and that surrogates are often motivated by altruism. Likewise, Ciccarelli and Beckman (6) argue that although most studies are methodologically inadequate, existing evidence points in the same direction: surrogates are motivated mainly by altruism, relationships between surrogates and intended parents are experienced as satisfying, and surrogates rarely have problems relinquishing the child. Teman’s (7) anthropological critical review of psychosocial research on surrogacy argues that the reviewed studies approach the issue from normative ideas about motherhood, family, and women. Teman calls for research to take surrogates’ own narrated experience at face value and suggests that this may lead to new approaches in research and important implications for policy. Busby and Vun (8) have conducted a qualitative review of 40 empirical studies on surrogacy in order to compare it with feminist critique of surrogacy and assess to what extent their ethical concerns match empirically documented outcomes. Also this review article suggests that common anxieties about exploitation and surrogate’s bonding with the baby are countered by existing evidence.

It should be noted that existing review articles disproportionally deal with research conducted in more affluent countries, where financial inequalities between surrogates and intended parents are generally less stark than in transnational surrogacy arrangements in poorer countries. It is reasonable to assume that this may have significant consequences for the results. In most reviews ethnographic research is excluded altogether. With its specific focus on how the insights from existing qualitative ethnographic- and interview-based research on surrogacy can contribute to debates on surrogacy, this review article addresses this lack.

## Beyond medical risks, psychological assessments, and individual choice: a relational approach to reproductive justice

The theoretical paradigm of *reproductive justice* seeks to go beyond both simplistic liberal notions of choice and all-encompassing structural ideas of exploitation, and can therefore offer a framework for more complex and nuanced analyses of surrogacy, taking into account the agency of surrogates, potential vulnerability of intended parents, and the moral issues that arise when surrogacy services are ‘outsourced’ to less affluent countries in the world (9,10). The concept of reproductive justice has been defined as ‘the normative concept that all women, regardless of their ethnic, racial, national, social, or economic backgrounds, should be able to make healthy decisions about their bodies and their families’ (11). Other researchers have argued for the need to extend the concept to include other groups that might be negatively affected by policies on reproductive rights, including HBTQ + persons (12,13), and to emphasise ‘intersectional social identities’ and the need for ‘community-developed solutions to structural inequalities’ (14). Drawing on a reproductive rights paradigm we argue that surrogacy is *intrinsically* neither ethical or unethical, but rather that its ethical status must be assessed contextually, depending on how well it is possible in a given context to: (1) negotiate potentially conflicting interests; (2) to secure reproductive rights and needs of all the involved parties in a satisfactory way; and (3) to build a relationship that is sustainable for surrogates, intended parents, and surrogacy-conceived children during and after conception, pregnancy, and birth.

For this to be conceptualized and possibly put into practice in legislation and policy, however, it is necessary to combine the more structural perspectives on reproductive justice with *a relational approach to ethics*. Relational ethics views persons not only as autonomous agents, but also as human beings involved in relationships. Each person is the centre of their own decision-making, but is at the same time related to others on different levels (family, social groups, society, etc.). This involvement in relationships influences individual decisions and embeds an understanding of justice in the context of human interaction.

Relational ethics is closely connected to feminist ethics and ethics of care (15). Some scholars make a distinction between a ‘causal view’ and a ‘constitutive view’ in relational ethics. The ‘causal view’ emphasizes the relational context of autonomous choices influencing individual decisions. The ‘constitutive view’ looks at persons as directly involved in relations, concerns, and care for others (16). Our approach is built on a constitutive view on relational ethics, and asks for justice in the context of the relationship between intended parents, the surrogate, and the surrogacy-conceived child.

## Method

We performed a critical interpretive literature review (17) specifically tailored to respond to bioethical inquiry, thereby emphasising relevance and quality of insights rather than ‘quantity for its own sake’. The review has been conducted in an iterative rather than a linear or stepwise process, using our initial research question flexibly, and continuously refining it in accordance with the results of the searches. Importantly, the quality and relevance of each article have been assessed in relation to how it can help responding to the qualitative and normative research question. This means that also articles that are not easily adaptable to simple assessments of methods have been included, such as ethnographic or mixed-methods studies, which allows for the inclusion of any potentially useful insights the study can bring. Only seriously flawed studies have been excluded (18).

With the research question in mind we included articles which: (1) focus solely or predominantly on surrogacy; (2) are based on ethnographic data and/or in-depth interviews with surrogate mothers and intended parents, preferably including substantial quotes from respondents’ own narratives (including some relevant studies of online forums or email interviews); and (3) include analysis of relations between surrogates and intended parents, and surrogacy-conceived children and other parties involved, such as clinic staff, doctors, and agents. To ensure that all studies included have undergone assessment of quality based on their own (rather than external) disciplinary norms, the sample has been limited to articles published in peer-reviewed international journals published in English.

Initial searches were broad, and included hundreds of articles, but with the use of keywords such as ‘surrogates’, ‘surrogate mothers’, ‘surrogacy’, ‘intended parents’, ‘interviews’, ‘ethnography’, ‘qualitative method’, and ‘relation(s)’ it was narrowed down to more than a hundred texts. Complementary searches were made to increase the geographical distribution of studies and to ensure a range of studies from different decades, spanning from early surrogacy practices in the USA in the 1980s and 1990s to recently published research. We ended up with a total of 39 articles that met all of our criteria. Articles were continuously read through carefully and were manually recorded in a table, including themes, quotes, and findings relevant for this review.

## Results

Selected articles belonged to a range of different disciplines, the majority of which from the interpretative human and social sciences (sociology, social psychology, anthropology, ethnology, and gender studies) and a smaller number from psychology, social work, or nursing. The number of interviews in each study ranges from as few as seven to over one hundred. Countries covered include Australia, Canada, Greece, India, Iran, Israel, Italy, Mexico, Norway, Russia, Sweden, UK, Ukraine, and the USA. Most studies focus only on surrogacy practices in one country (although often with intended parents from other countries), and some include several countries (e.g. interviewees from several countries or fieldwork in different field-sites).

Many studies (some with overlapping samples) focus solely or mainly on surrogacy practices in a single country, e.g. thirteen on Indian (19–31), nine on the US (32–40), and five articles on the Israeli context (36,41–44). The least studied country contexts in this sample include Greece (45), Iran (46), Italy (47), Mexico (48,49), Norway (50), Sweden (51,52), and Russia/Ukraine (53,54). In countries where surrogacy is not legal, only intended parents through transnational surrogacy have been interviewed (e.g. Italy, Norway, Sweden), and some studies have interviewed intended parents or surrogates in several countries (55,56). The vast majority of studies investigate commercial surrogacy arrangements, but some investigate altruistic surrogacy. These have either been conducted in countries such as the UK and Greece where only non-paid surrogacy is permitted, or have been specifically designed to investigate altruistic surrogacy drawing on interviews with surrogates in several countries (56). In addition, there are some examples of this in Berend’s online ethnography (32–35). Most studies concern gestational surrogacy and focus exclusively or mainly on heterosexual intended parents, with only few including same-sex couples and non-heterosexual individuals (40,44).

## Characteristics of the relation between surrogates and intended parents: closeness, expectations, and satisfaction

The following analysis has been conducted in three steps. First, we identified four frequently occurring themes or ‘areas of negotiation’ in the literature, that is, areas of real or imagined potential tension or difficulty that is resolved differently in the different samples and contexts. Second, we have constructed a table of ‘ideal types’ of surrogacy relationships, based on the empirical findings. Third, we analysed the implications of our findings for future research, ethical considerations, and policy debate.

### To bond or not to bond: varieties and strategies of proximity and distance

The first theme concerns the existence (or non-existence), creation, or dissolution of *bonds* between surrogate and intended parents, between surrogate and child, and between intended parents and child. This was significantly affected not only by geographic proximity or distance but also through the creation of emotional closeness or personal boundaries.

In some cases, relationships between surrogate and intended parents were very close; especially intended mothers were very engaged throughout the pregnancy—coming to pregnancy appointments, birth classes, and delivery, often ‘managing the pregnancy’ by being in charge of medical aspects. In these relationships, both surrogates and intended mothers would engage in emotional work to define the pregnant belly as an extension of the intended mother. Surrogates would often ‘transfer’ pregnancy symptoms to intended mothers by reporting on them (e.g. phoning when feeling nausea). Some intended mothers reported a ‘pseudopregnancy’ or (psycho)somatic symptoms such as gaining weight or the secretion of colostrum or feeling contractions when the surrogate went into labour (1,42). While surrogates are continuously reported to use strategies or mental frames to separate the pregnancy and foetus from themselves (e.g. to prevent bonding or to communicate never having felt a bond), there are examples of intended parents (mainly mothers) who wear a ‘dummy belly’ or record their voice for the surrogate to play to the foetus in order to aid the bonding process (23). In some cases, physical proximity and frequent contact were used as a means to control the surrogate (diet, exercise, etc.), especially in cases where she was living together with the intended parents or in-laws (25,42).

In studies where geographical distance was larger, bonds could be created and upheld over the phone or through online communication (27,33,39,47,57). Choosing a distant country for surrogacy is sometimes deliberate: it may be a way to distance oneself from the surrogate and strengthen the borders of the newly established nuclear family (47). In stark contrast, Israeli gay couples interviewed by Ziv and Freund-Eschar (44) experienced significant frustration and anxiety over lack of contact with surrogates, describing an ‘abstract’, ‘theoretical’, and ‘disembodied’ pregnancy, and expressed feelings that the pregnancy consisted of documents, was lacking in emotions, and that they missed being closer to the surrogate so as to bond with the child (e.g. stroke belly).

### Related or not related: the use of relationship and kinship vocabularies

The second theme concerns the *kind of relationship* between surrogate and intended parents as well as the one between the surrogate and the child. We found that these are often negotiated through identification or disidentification using a family, kinship, or kinship-like vocabulary.

Although surrogates, most notably in the US and Israeli studies, express clearly that they are not related to the baby, some differences in emphasis were detected. In the US studies, parentage was more often described in terms of intentions, while in Israel the genetic link to intended parents was foregrounded (32–35). Although these surrogates would generally denounce any kinship to the child, for example by likening themselves to an ‘oven’, ‘kindergarten teacher’, ‘nanny’, or ‘babysitter’ (35,53), in other studies kinship metaphors were used to describe the surrogate’s relationship to the child, like ‘auntie’ (35,47) or ‘tummy mommy’ (55), or exceptionally even ‘mom’ (23). In the Indian context there are several examples of surrogates expressing a non-genetic kinship relationship to the children they carried referring to ‘blood, sweat, and milk’ (standing for both work and substances) to claim kinship or kinship-like relationships or expressing how they would miss ‘their babies’ (21,22,27). In contexts where a close relationship between surrogates and intended parents is the norm (UK, USA), kinship and family vocabulary is used to describe the relationship, e.g. that they are ‘*like* family’ (e.g. 35,39). In other contexts, the family bond is resisted and kinship metaphors are rarely used (35). In the Indian context, the metaphor of surrogates and intended mothers being ‘sisters’ frequently recurs (20–23,25,27).

### Context: culturally available narratives, norms, expectations, and disappointments

The third theme concerns how *norms and expectations* of the surrogacy relationship are differently constructed and interpreted in different contexts through *culturally available narratives*. Significantly, the correspondence or discrepancy between these narratives and the experienced reality seems to impact satisfaction and disappointment.

More or less idealist narratives of surrogacy as a mutual ‘journey’ undertaken by surrogates and intended parents are widespread in some contexts where relationships are more equal (32–35,43,56). In Berend’s US study, romantic metaphors were used, including language from dating and falling in love (e.g. about finding the ‘perfect couple’, ‘the ones’, ‘we clicked’) (33). Even though strong similarities in narration have been identified in earlier studies (1), in contexts where surrogates and/or intended parents form online communities, narratives and norms have been shown to become more standardized, sometimes almost identical. A romanticized narrative may also work as an incentive for women to become surrogates, constructing a norm against which their own experience is measured, which can lead to disappointment (43). The reviewed studies include evidence of how surrogates consistently feel disappointment or even grief when the relationship with the intended parents does not live up the expectations. Surrogates express feeling ‘ditched’, ‘tossed aside’, and betrayed when a close relationship does not materialize, fades, or deteriorates during or after pregnancy (36), or if the intended parents turn their backs in case of failed treatments or pregnancy loss (32).

Conversely, more precautionary cultural narratives of the surrogacy relationship can lead to distrust and are sometimes used to legitimate monitoring by agencies. These include media narratives that warn about surrogates who do not want to relinquish the baby and intended parents who do not want to claim the child, especially in case the child is ill or disabled. In other cases, precautionary narratives are cultivated by agencies and intermediaries who warn intended parents of ‘greedy surrogates’ (25,48), thereby strengthening their own position and ability to fully control the relations between them and the surrogates. In some other countries, such as Russia, surrogates prefer to frame their relationship as business-like and based on legal regulations and monetary exchange, rather than trust and emotional closeness. Such a narrative, as Siegl (53) has observed, leaves little room for expressing feelings, which in turn may cause distress when emotions might be felt anyway.

### ‘The gift of life’ or monetary transaction: conceptualising the exchange

The fourth theme concerns the coexistence and sometimes tension between the idea of surrogacy as a form of *‘gift-giving’ or as a business transaction*. The relationship between altruism and monetary exchange is complex and is negotiated in a variety of ways across different contexts. Among Swedish intended parents the surrogacy relationship is described as a ‘win–win’ situation (e.g. surrogate gets money, intended parents receive their long-awaited child) (51). Money might be described as a motive simultaneous to altruism, and/or affective narratives are used to downplay the economic exchange (34,40). In some contexts, such as India, expectations of extra-contractual gifts were reported (25), along with intended parents’ discomfort by being reminded of the surrogate’s poverty or requests for additional remuneration (48,49). Money, however, tends to be symbolically charged in different ways, and there are many clear examples where paid surrogates do not want the relationship to be reduced to a business transaction, and cases where they retroactively regret not charging the intended parents what they felt they were entitled to, when their emotional needs were not fulfilled (32,33).

Narratives of altruism are frequently used by agencies in advertising, and in some countries such as India and Ukraine the agency controls communication between surrogates and intended parents to avoid surrogates requesting more money (54). The Russian case, where ideals of the surrogate as an entrepreneur or business person are conveyed in the interviews with surrogates (53), appears to differ significantly from other contexts. This tendency can, however, be interpreted as yet another strategy to avoid stigma and manage conflicting emotions on the part of surrogates.

## Analysis

Based on the outcomes of the studies included in this review, we propose a typology of relations between surrogates and intended parents. Four types are identifiable: *open*, *restricted*, *structured*, and *enmeshed* (Table 1). The types take into account criteria which influence these relationships, such as frequency and character of contact pre-and post-birth; expectations of both parties; the type of exchange involved in surrogacy arrangements; as well as the cultural, legal, and economic context.

**Table 1.** Tentative typology of relations between surrogates, intended parents, and child.

Type of relation	Characteristics of contact	Expectations towards surrogacy	Exchange / what is being exchanged	Context
Open	Regular, caring, intense, also spontaneous. Face-to-face at least a couple of times, with few or no intermediaries.	Fulfilled, high level of satisfaction on both parties.	Emotions: love, joy; solidarity, friendship, sense of shared experiences, as well as money and material gifts.	Mostly (but not exclusively) emerging in contexts where power disparities are relatively low.
Restricted	No contact or sporadic contact, although in some cases frequent contact if surrogate lives with intended parents during pregnancy.	Low expectations, some surrogates are not interested in relationship, but others report that they would need more contact and support during pregnancy, while intended parents are withdrawn or absent.	Focus on monetary compensation and low expectations as to other aspects of exchange; no space for re-negotiating the terms.	Defined mostly by power inequalities, structural conditions of surrogacy industry in countries of the Global South or the post-communist region, but also reinforced by diverging cultural narratives.
Structured	Planned at regular intervals: more often pre-birth, occasional post-birth (postcard or a phone call from time to time).	Fulfilled to a large extent, based on contractual frames, relatively low expectations as to emotional and relational aspects of surrogacy.	Surrogacy is structured as labour, which needs to be compensated; limited possibilities for re-negotiations.	Dependent on the cultural scripts, a big role of legal contract for this type of relation.
Enmeshed	Characterized by tendency to expand the boundaries established in contract or expected by one party, contact is often intense in frequency or emotional content.	Unfulfilled or failed, due to divergent expectations and/or different cultural scripts regarding parenthood and surrogacy.	Diverging visions of what is being exchanged: surrogate focuses on ‘gift of life’ and assumes that obligations of intended parents go beyond contract, whereas intended parents either do not understand or do not accept any emotional or other extra-contractual obligations.	Mostly contexts characterized by power inequalities, where low compensation for surrogate accompanies cultural narratives around surrogacy as a precious gift, but it can also occur in other contexts.

### Open relationships

Open relationships are characterized by *regular, caring, and intense contact* between the intended parents and the surrogate. The frequency depends mostly on conditions, e.g. geographical proximity, but is often spontaneous, initiated ad-hoc. Face-to-face contact often occurs at least a couple of times during pregnancy, which may be initiated by both surrogate and intended parents. This type of relationship is marked by emotional openness and direct contact with few or no intermediaries between the reproductive parties. The intended parents are usually present at birth, and the surrogate sometimes maintains regular contact with the child/parents after birth:

We wanted one [surrogate] who was very open … this summer [we] spent one week with them in California. And the kids Skype with her frequently. And they call her tummy mummy. (55)

In open relations there is a *matching level of expectations* of both parties as to the development of the relationship, thus the expectations of both parties are usually fulfilled and reported levels of satisfaction high. Close contact with intended parents may help the surrogate to focus her emotional bond on the intended parents rather than the baby:

It is an amazing feeling to find the PERFECT COUPLE … ahhh, the romance, I miss it! (33)

In some situations, it is expected that the surrogate will develop a relationship with intended parents and the child as a friend or even relative.

We bond more with the couples than the babies […] Our friendship doesn’t end at birth; hopefully it grows into more of a family bond than what’s already there! (35)

[T]hey are like family to us … (39)

What is being exchanged between the reproductive parties is described as emotions such as love, joy, solidarity, friendship, and sense of shared experiences, as well as material gifts:

[It’s] a journey of shared love … (33)

[T]hey’d be there for me anytime I need them and I’m there for them and yeah I can confide in them … they’re good friends … (57)

They visit me weekly and every time they bring biscuits, fruits, and dry fruits. … They really love me a lot. (27)

Open relations emerge mostly (but not exclusively) in contexts where power disparities are relatively low, and where cultural narratives of surrogacy tend to match the expectations and socio-economic status of the surrogate. In case of altruistic surrogacy it is measured mostly in a close, nurturing relationship with the intended parents, and in the case of commercial surrogacy it is a combination of security, financial gratification, and emotional support according to individual needs (see also 58).

#### Restricted relationships

In contrast, in restricted relationships there is normally *no contact or sporadic contact between the surrogate and the intended parents*, although in some cases (e.g. in India) frequent contact occurs if the surrogate lives with intended parents during pregnancy. Often, there is very limited or no face-to-face contact pre-birth, and just one meeting during delivery. Such relationships are mostly or fully controlled by the intermediaries (agency, doctors) and/or intended parents:

[M]y first contact was through video call when I was two months pregnant, and I only met them personally when the baby was born … (49)

Restricted relationships are characterized by low expectations as to the quality of contact, especially on the part of the surrogates. Some surrogates report not being interested in a continuous relationship as ‘their work ended with the delivery’ (35), but others claimed that they would need more contact and support during pregnancy, while intended parents were withdrawn or absent:

Being in contact with the parents would help her emotionally, she says, because then it would feel much clearer that the child is not hers—now, without the contact, it instead feels like she is carrying her own child. (53)

Lack of contact was also problematic for some intended parents, who spoke of how such an ‘abstract pregnancy’ made it difficult for them to prepare mentally for parenthood (44). For surrogates, restricted relationships might be especially traumatic in case of failed pregnancy:

When intended parents walk away as if from a failed transaction, they deny the value of the surrogate’s gift and the magnitude of her sacrifice. (32)

Restricted relationships are framed by a focus on monetary compensation and low expectations as to other aspects of surrogacy. Surrogates often reported that their needs and expectations remained unfulfilled, because intended parents often saw the surrogate just as a ‘vessel’ or a hired labourer, as expressed in the following quote:

… I did not find that lady [previous surrogate] right. Her words and demeanor was not very convincing after the unsuccessful conception. (26)

This type of relationship increases the probability that the surrogate would regret her decision to become one:

Most of the surrogates in the study spoke of their distaste in doing what they had done or where undertaking, saying that they would not do it again and had been forced to do it by the circumstances. (25)

The development of restricted relationships is defined mostly by already-existing power inequalities and structural conditions of the surrogacy industry, for example in countries of the Global South or the post-communist region. They can also be reinforced by diverging cultural narratives, when the elevated status of the surrogate as the giver of the precious ‘gift of life’ is not matched by good conditions, monetary compensation, and/or appreciation and support. This type of relationship reflects highly unequal power relations and status between the reproductive parties and was not reported in studies on altruistic surrogacy.

#### Structured relationships

Structured relationships are highly contingent on pre-existing conditions, often described in the contract. Such contracts usually include contact planned at regular intervals: more often pre-birth, ocasionally post-birth, a postcard or phone call from time to time. In practice, these may mean a couple of meetings, either face-to-face or internet-mediated, initiated by the surrogate or the intended parents. Often the meetings are mediated through an agency, but not strictly controlled.

Contact before and after the birth is regulated by the contract, including: (1) lifestyle rules and behavioural restrictions; (2) rules governing breastfeeding, and ‘intimate’ form of contact with the baby; and (3) rules governing viewing, handling, and future relationships with the newborn (37). The expectations of both parties are often fulfilled to a large extent, based on contractual regulations:

I’m satisfied, it’s what we wanted. If she felt she wanted more contact we’d definitely do it more […] I think we’re on the same page. (47)

What do I need this personal contact [with intended parents] for? I do my job and get money for it; that’s all I need to know. (53)

Dependent on the cultural scripts, e.g. preference for a business-like relation by Russian and Ukrainian surrogates (53,54), it also reflects a strategy on part of the surrogate to mediate social stigma attached to surrogacy in most cultural contexts, and facilitate emotional detachment in relation to the baby.

Within the framework of structured relations, surrogacy is seen as a form of labour which needs to be compensated, and there is a big role of the legal contract for the ways in which it is organized. The more inclusive it is of unexpected and changing needs, the more possibility there is for the surrogate to get the support she needs. Both parties tend to report that their formal expectations were fulfilled, but these are often relatively low with regard to emotional and relational aspects of surrogacy. Sometimes emotional expectations change over the course of or after pregnancy, but there is very little or no room for negotiations:

She is my first baby girl. I have two sons and I always craved for a girl. I know she looks Japanese but I think of her as my own daughter. [intended mother agreed that she was] on my breast for two whole months. … I miss my daughter, you don’t know how much. (23)

#### Enmeshed relationships

The last type of relation is the enmeshed one characterized by a tendency to expand the boundaries established in the contract or expected by one party, as well as intensive contact both in terms of frequency and accompanied emotions. The surrogate and the intended parents may get in touch face-to-face and through social media. Sometimes contact continues with increasing frequency and/or intensity post-birth, generally initiated by the surrogate rather than intended parents. Relationships between the parties are often controlled via intermediaries but only to a certain point, as there is usually no mediation post-birth:

Clinics and agencies encourage [surrogates to demand] informal payments [post-birth] to offset the low compensation they offer to the surrogate. (25)

The expectations of both intended parents and surrogates are often unfulfilled or failed, due to divergent expectations and/or different cultural scripts:

I used to think they would invite us to America. I used to think of her as a sister—all of it went to waste. Forget an invitation, they did not even call to see if we are dead or alive. (23)

The practice of gift-giving [in India] also follows a particular form of obligatory logic, wherein the surrogate seeks compensation through gift and extra contractual money from the intended parents. (25)

This type of relationships occurs mostly in contexts characterized by power inequalities, where low compensation for surrogates accompanies cultural narratives around surrogacy as a precious gift, e.g. India or Mexico. Such outcomes, however, can happen also in other contexts, e.g. the USA, when expectations prior to the surrogacy arrangement do not meet the quality of the relationship with intended parents during pregnancy or after birth. The surrogate focuses on ‘gift of life’ and assumes that obligations of the intended parents go beyond contract, whereas the intended parents either do not understand or do not accept any emotional or other extra-contractual obligations, even though they may eventually give in to requests:

I have paid the surrogate a lot of money. I want her to understand that we are done now. She cannot keep on asking for more. (49)

[H]ere I am doing something for you and you treat me like you are scared of me … (39)

I went through so much physically and emotionally for them to have a family and … they vanish into the great unknown like a stranger in the grocery store. (32)

## Conclusion

Our analysis highlights the ways in which the framework of relational justice can inform bioethical and policy debates. We point to the importance of a longitudinal perspective to surrogacy, and the need for recognizing changing expectations and needs of the people involved. Whereas new conceptualizations of parenthood increasingly recognize that people having no genetic or gestational connection to children are part of an extended kinship network (e.g. ‘bonus parents’ in patchwork families), in the case of surrogacy there is a tendency to perceive the surrogate as either ‘the mother’ (or someone who wants to keep the child) or just a ‘vessel’ or ‘gestational carrier’.

Existing research shows that while most surrogates do not identify as mothers, they often wish to sustain contact with intended parents and children, and such contact is key to emotional stability and satisfaction. Thus, there is a need for an informed debate and policies taking into consideration the possibility for promoting sustainable relations between the parties involved in surrogacy arrangements over a longer period of time. This requires a certain amount of flexibility and open-endedness inscribed in both legal ramifications and everyday practices, which should be adjusted for the evolving needs and emotional perspectives pre- and post-birth, also in case of failure at any given stage of the procedure. The possibility for including a relational justice perspective, however, depends largely on the intermediaries, especially the agencies managing commercial surrogacy, as well as non-governmental organisations helping in altruistic arrangements and state institutions. The key challenge for these actors is to recognize the temporal changes and open-endedness of the relationship between the surrogate and intended parents, taking more responsibility not only for financial and medical risk management but also for the quality of relational aspects of surrogacy arrangements over longer periods of time.

Adequate medical and psychological evidence is imperative for ethical debates and policy discussions on the issue, but we need to recognize that controversies are intimately tied to issues of values that go far beyond scientific facts. Surrogacy cannot be detached from cultural meanings and ideological ideals concerning motherhood/parenthood, kinship, rights and justice, fairness and equality, and ideas of agency and ‘free choice’. There is simply no ‘neutral’ way of approaching the issue. Considering this complexity, we conclude that it is necessary to develop a more nuanced approach to surrogacy, one that includes a relational justice approach and merges deep knowledge of cultural norms and socio-economic inequalities in the local context with recognition of both the agency and potential vulnerabilities of surrogates and intended parents alike.
